# Random Mutagenesis in *Porphyromonas gingivalis* Enables Isolation of Mutants With Enhanced Secreted Protease Activity

**DOI:** 10.1111/omi.70024

**Published:** 2026-03-04

**Authors:** Takeru Nakabayashi, Satoshi Yuhara, Kosei Tanaka

**Affiliations:** ^1^ Department of Research Testing Development H.U. Group Research Institute G.K. Akiruno Tokyo Japan; ^2^ Innovative Research Department H.U. Group Research Institute G.K., Akiruno Tokyo

**Keywords:** periodontal disease, *rgpA* gene, secreted protease, virulence factor

## Abstract

*Porphyromonas gingivalis* is a key pathogen in periodontitis, with secreted proteases as major virulence factors. We developed a screening method to generate and identify *P. gingivalis* mutants with elevated protease activity. Mutations were induced using the mutagens 2,6‐diaminopurine (2,6‐DAP) or ethyl methanesulfonate (EMS), and the mutagenized cells were subsequently plated on casein agar. During colony growth, the medium became opaque due to partial casein precipitation, whereas colonies with higher protease activity produced clear halos through casein degradation. Colonies that formed halos earlier than the wild type were selected for further analysis. Liquid culture assays of the supernatants identified four strains with enhanced protease activity, of which two were 2,6‐DAP‐derived and two were EMS‐derived. Whole‐genome sequencing revealed that the two 2,6‐DAP‐derived strains carried mutations in iron transport‐related genes (*foeA* and *tonB*, respectively), likely increasing protease levels through iron limitation–induced upregulation of *rgpA*. The two EMS‐derived strains contained multiple mutations, including one in *rgpA*, a major protease gene. The N‐terminal region of RgpA, which contains the protease motif, harbored the G450D mutation in one strain and the C600Y mutation in the other. These results demonstrate that our method efficiently generates *P. gingivalis* mutants with protease gene alterations that increase enzymatic activity. This approach provides a useful tool for studying protease function and virulence mechanisms in this pathogen, and for identifying genes that affect protease secretion.

**Abbreviations**
2,6‐DAP: 2,6‐diaminopurine
EMS: ethyl methanesulfonate

## 1 Introduction

Periodontitis is a chronic inflammatory disease characterized by the progressive destruction of the supporting tissues of the teeth, including the gingiva and alveolar bone (Xu et al. 2020). The subgingival environment harbors a complex microbial community dominated by anaerobic bacteria. Among the species associated with periodontitis, the so‐called “red complex”—comprising *Porphyromonas gingivalis*, *Tannerella forsythia*, and *Treponema denticola*—has been strongly implicated in disease pathogenesis (Holt and Ebersole 2005; Jiang et al. 2021). Within this group, *P. gingivalis* is recognized as a key pathogen that shapes the subgingival microbial community and promotes pathogenic processes (Bostanci and Belibasakis 2012). Its interactions with other microbes and the host create conditions that favor chronic inflammation and tissue destruction, highlighting the importance of its virulence factors (Hajishengallis et al. 2011).

Among these factors, FimA, extracellular polysaccharides (EPS), lipopolysaccharide (LPS), and outer membrane vesicles (OMVs) have been identified as virulence factors (Lunar Silva and Cascales 2021); however, gingipains are recognized as major virulence‐associated proteases of *P. gingivalis* (Potempa et al. 2003). Gingipains are secreted cysteine proteases comprising three enzymes: arginine‐specific RgpA and RgpB, and lysine‐specific Kgp. They cleave diverse host proteins through their catalytic domains, representing the major proteolytic enzymes of *P. gingivalis* (Potempa et al. 2003). Through these activities, they degrade major components of the periodontal extracellular matrix, such as collagen and fibronectin, and disrupt cell–cell and cell–matrix adhesion (Jia et al. 2019; Ruggiero et al. 2013). They also degrade hemoproteins and transferrin to acquire iron and heme, which are essential for bacterial growth and survival (Sroka et al. 2001). These proteolytic activities contribute to local tissue destruction and disease progression, and they have also been implicated in systemic conditions such as atherosclerosis and rheumatoid arthritis (Hajishengallis 2015). To elucidate the mechanisms underlying these activities, structural and biochemical studies have investigated the organization of gingipain enzymes.

Among the gingipains, RgpA and RgpB are highly similar enzymes, with RgpB often studied as a model for structural and functional analyses. RgpB is composed of a catalytic domain and a pro‐domain. The pro‐domain, present in the zymogen form of the enzyme, contains an autoinhibitory loop that regulates both enzymatic activity and substrate specificity (de Diego et al. 2013). Despite these insights, the precise amino acid residues or regions that quantitatively determine protease activity, as well as the potential existence of previously unreported gingipain‐like proteases, have yet to be fully elucidated. Understanding these determinants is essential for elucidating the molecular mechanisms underlying *P. gingivalis* virulence.

In the present study, we aimed to establish an efficient screening method to isolate *P. gingivalis* mutants exhibiting increased protease activity and to identify the genetic determinants responsible for the observed phenotypic changes. This approach provides a valuable tool for dissecting gingipain function and understanding the molecular basis of *P. gingivalis* pathogenicity.

## 2 Materials and Methods

### 2.1 Bacterial Strains and Growth Conditions

*Porphyromonas gingivalis* ATCC 33277, the type strain used as the wild‐type (WT), and derived mutant strains were used in this study. mGAM liquid medium (AccuDia GAM Broth Modified, Shimadzu) at 41.7 g/L, autoclaved, was used for cultivation, with 15 g/L agar added for solid medium preparation. GCH agar medium, composed of 0.2× mGAM liquid medium, 20 g/L casein sodium, 15 g/L agar (autoclaved), and supplemented with 2 mg/L hemin (prepared by adding 0.4 mL of 5 g/L hemin dissolved in 1 M NaOH and sterilized by filtration), was used for the selection of high‐protease‐activity strains. Cultivation was performed at 37°C in an anaerobic rectangular jar (Mitsubishi Gas Chemical Co., Inc.) with an anaerobic sachet (Mitsubishi Gas Chemical Co., Inc.). To induce random mutation in *P. gingivalis*, 2,6‐DAP was added to a final concentration of 100 µg/mL (from a 1 mg/mL stock solution, sterilized by filtration) or EMS was added at 10 mM, and cultures were grown from an initial OD_600_ of 0.01 until reaching approximately 1.0.

### 2.2 Selection of Strains With High Protease Activity

Although 2,6‐DAP and EMS are classical mutagenesis agents, they remain useful for inducing random point mutations in strains where targeted genetic tools are limited. We employed this strategy to obtain unbiased mutants potentially affecting protease activity without prior assumptions about candidate genes.

*P. gingivalis* cultures treated with mutagens such as 2,6‐DAP or EMS were spread on standard 90‐mm diameter GCH agar medium at a density of approximately 500 colonies per plate. The inoculum was adjusted to allow well‐separated colonies suitable for individual isolation. The GCH agar medium was originally transparent, and colonies became visible around day 6 after spreading. As the colonies grew, the surrounding medium became opaque due to partial precipitation of casein. After further incubation, colonies forming clear halos around them were identified, and these halo‐forming colonies were selected as candidate high‐protease‐activity strains.

### 2.3 Secreted Protease Activity Assays

Strains were pre‐cultured in mGAM liquid medium for 3 days. Log‐phase cells were diluted to an OD_600_ of 0.005 in fresh mGAM medium and incubated for 24 h in three biological replicates. After cultivation, OD_600_ was measured to confirm that the cultures were in the mid‐log phase, and 1.0 mL of culture was then collected. Cells were removed by centrifugation at 14,000 × *g* for 1 min, and the supernatant was used to assess protease activity. Supernatants were diluted 20‐fold, and protease activity was determined according to the manufacturer's instructions using the Red Protease Detection Kit (Sigma‐Aldrich) with a BioTek Synergy H1 Multimode Reader (Agilent). Protease activity was normalized to an OD_600_ of 1.0, and the activity of each strain was expressed relative to the simultaneously measured WT.

### 2.4 Identification of Mutations

DNA of strains was extracted using the ISOSPIN Fecal DNA Kit (NIPPON GENE) and the FastPrep‐24 5G homogenizer (MP Biomedicals). Sequencing libraries were prepared using the NEBNext Ultra II FS DNA Library Prep Kit for Illumina and the NEBNext Multiplex Oligos for Illumina (Dual Index Primers Set 1) (New England Biolabs) according to the manufacturer's protocol. The libraries were size‐selected to an average fragment length of approximately 700 bp, and the fragment size distribution was confirmed using the TapeStation D1000 (Agilent Technologies). The libraries were sequenced on the MiSeq sequencer (Illumina) using the MiSeq Reagent Kit v2 for 250‐bp paired‐end reads.

Reads were quality‐trimmed using fastp (v0.23.2) with default parameters, and then mapped to the corresponding reference sequence (*P. gingivalis* ATCC 33277, NCBI RefSeq assembly: GCF_000010505.1) using the Burrows–Wheeler Aligner (BWA‐MEM, v0.7.17) with default parameter.

Variant calling was performed using GATK (v4.6.1.0) with the ‐ploidy 1 option. For minor variant detection, the ‐ERC GVCF option was additionally applied.

Variants with read depth < 10 were excluded. The resulting variant lists, including genotype information in the format GT:AD:DP:GQ:PL (where GT = genotype, AD = allele depth, DP = read depth, GQ = genotype quality, and PL = phred‐scaled likelihood of genotypes), are summarized in the tables.

## 3 Results

### 3.1 Random Mutagenesis and Selection of High‐Protease Activity Strains

**FIGURE 1:**
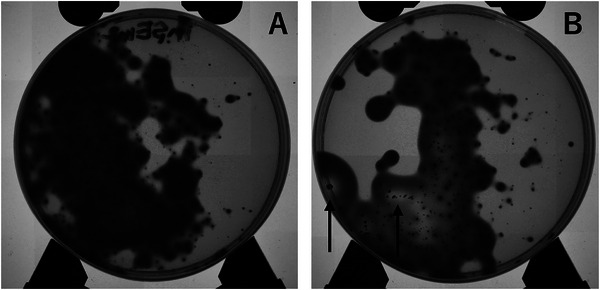
Formation of halos by *P. gingivalis* on GCH agar plate. Representative plates photographed under transmitted light. (A) Without halo formation. (B) Showing halo formation around colony clusters, indicated by arrows.

To induce random mutations in *P. gingivalis*, we treated the cultures with 2,6‐DAP or EMS, both of which have been widely used for mutagenesis in bacteria (Kalle and Gots 1961; Sobotková et al. 1988). The mutagen‐treated cultures reached an OD_600_ approximately half that of the untreated control. To select mutants with elevated secreted protease activity, the cells were plated on GCH agar medium. Colonies appeared approximately 6 days after plating. As *P. gingivalis* did not form colonies on 0.2 × mGAM agar plates (GCH agar medium without casein), it is likely that casein in the GCH agar medium served as a major nutrient source, with nutrients gradually supplied through secreted protease‐mediated degradation of casein. Indeed, *P. gingivalis* has been reported to utilize amino acids as energy and carbon sources (Nemoto and Ohara‐Nemoto 2016). The GCH agar medium was initially transparent. As colonies grew, partial degradation of casein caused the medium to become opaque, as the degraded casein precipitated and formed an opaque zone (Figure 1A). This observation is consistent with results in mC medium (Sasaki et al. 2025), in which secreted proteases induce turbidity, suggesting that the opacity in GCH agar medium arises through the same mechanism. In some colonies, continued cultivation gradually cleared the opaque zone surrounding the colonies (Figure 1B). Colonies that cleared earlier than the wild‐type strain were selected as candidate high‐protease‐activity strains, and their secreted protease activity was subsequently quantified.

### 3.2 Measurement of Secreted Protease Activity

**FIGURE 2:**
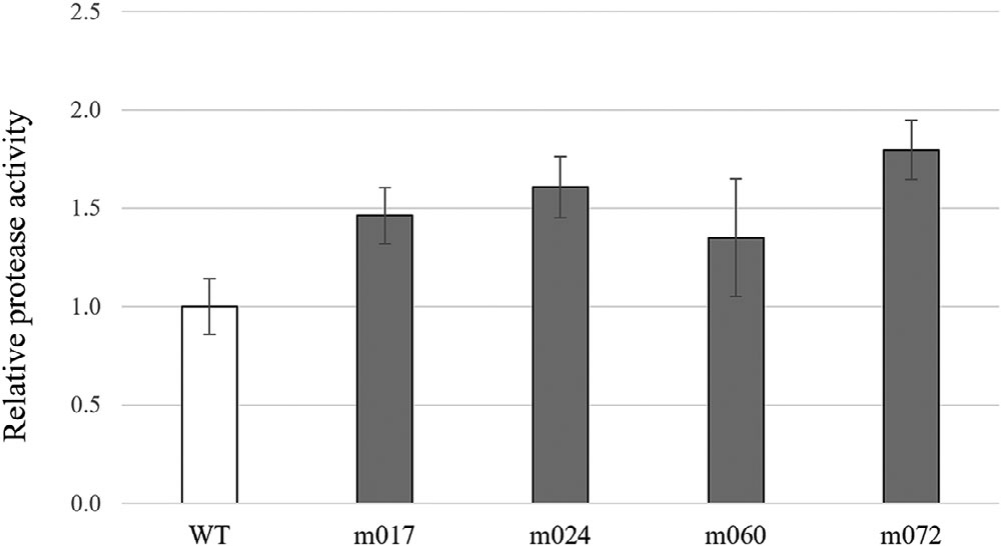
Protease activity of *P. gingivalis* mutants with elevated activity. Protease activity was measured using the Red Protease Detection Kit and normalized to an OD_600_ of 1.0. Activities are shown relative to the wild‐type strain. The white bar represents the wild‐type strain, and the gray bars represent mutant strains. Data are shown as mean ± standard deviation of three biological replicates.

Candidate strains with potentially high protease activity were cultured in mGAM liquid medium, and culture supernatants were collected at the logarithmic growth phase. Protease activity was quantified using the Red Protease Detection Kit (Sigma‐Aldrich), normalized to OD_600_ = 1, and expressed relative to the wild‐type strain measured in parallel. Using this assay, four mutant strains (m017, m024, m060, and m072) were identified as exhibiting higher protease activity than the wild type, with all four showing at least 1.2‐fold higher activity (Figure 2). Of these, m017 and m024 were generated using 2,6‐DAP, whereas m060 and m072 were generated using EMS.

### 3.3 Identification of Mutations

DNA sequencing was performed on four high‐protease strains and the wild‐type strain using MiSeq. The reads were mapped to the reference genome (NCBI RefSeq assembly: GCF_000010505.1). All strains showed an average coverage above 50, providing sufficient data for reliable analysis (Table 1).

**TABLE 1:** Mapping results of sequencing reads to the reference genome.

Strain	Coverage	SD
WT	57.80	13.49
m017	55.44	16.59
m024	73.36	16.41
m060	61.79	13.18
m072	52.63	11.86

*Note*: Average coverage and standard deviation (SD) are shown.

For the wild‐type strain, 27 variants relative to the reference genome were identified after excluding positions with coverage below 10 (Table S1). Examination of these variants in the high‐protease strains revealed that the C→A mutation at position 397119 was absent in strain m024. The heterozygosity of this mutation in the wild‐type strain (37/47) suggests that m024 derived from a population lacking this mutation. All other variants were consistently detected across the four strains (data not shown).

Next, mutations induced in the four mutant strains were identified. In the 2,6‐DAP‐derived mutants (m017 and m024), one mutation was detected in each strain, affecting PGN_RS05195 (*feoB*) and PGN_RS03865 (*tonB*), respectively—both genes involved in iron uptake (Table 2). These mutations may have affected iron transport efficiency, which could indirectly influence protease activity. EMS‐derived mutants (m060 and m072) contained 37 and 27 mutations, respectively. Two or three representative mutations from each strain are shown in Table 2, while all identified mutations are listed in Table S2. Several mutations were identified in intergenic regions. Some were located upstream of annotated genes and may reside within putative promoter or regulatory regions; however, no clear association with genes directly involved in protease production or secretion was identified.

**TABLE 2:** Summary of variants detected in the four mutant strains.

Strain	Total number of mutations	Genome position	Reference allele	Alternate allele	Genotype	CDS annotation	Amino acid changes	Gene function
m017	1	1209095	G	A	1:0,41:41:99:1419,0	PGN_RS05195	G593D	iron ion transport
m024	1	888976	G	A	1:0,71:71:99:2441,0	PGN_RS03865	A197V	energy transducer TonB
m060	37	917307	C	T	1:0,51:51:99:1737,0	PGN_RS03970	D2281N	SprA (Sov), T9SS core component
		1875711	C	T	1:0,64:64:99:2016,0	PGN_RS07990	A108T	TolC family protein
		2207874	C	T	1:0,14:14:99:482,0	PGN_RS09325	G450D	Arg‐gingipain RgpA
m072	27	314283	G	A	1:0,53:53:99:1853,0	PGN_RS01395	V49M	minor fimbrial subunit Mfa5
		2207424	C	T	1:0,58:58:99:2117,0	PGN_RS09325	C600Y	Arg‐gingipain RgpA

*Note*: Variants identified in the four strains are listed with their genomic positions, reference and alternate alleles, predicted amino acid changes, and gene function. Reads were quality‐trimmed using fastp (v0.23.2), mapped to the reference genome using BWA‐MEM (v0.7.17), and variants were called using GATK (v4.6.1.0) with the ‐ploidy 1 option, as described in Section 2.

**FIGURE 3:**
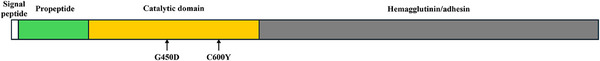
Domain structure of RgpA showing major regions along the amino acid sequence. Features are based on UniProt entry B2RM93 (CPG1_PORG3) and Peng et al. Positions of amino acid substitutions identified in our mutational analysis are indicated by arrows (corresponding to G450D and C600Y described in the main text). Major regions: signal peptide: 1–20, propeptide: 21–224, catalytic domain: 225–719, hemagglutinin/adhesin: 720–1703.

In contrast, mutations in RgpA, a major gingipain protease of *P. gingivalis*, were of particular interest. The detected mutations (G450D and C600Y) were located within the catalytic domain (residues 225–719) of full‐length RgpA (1703 amino acids), as shown in Figure 3, suggesting that these mutations might have contributed to the enhanced protease activity observed (Peng et al. 2020).

## 4 Discussion

### 4.1 Selection of Mutants With High Protease Activity

In this study, we aimed to obtain *P. gingivalis* mutants with elevated protease activity by introducing random mutations using chemical mutagens, a classical approach for mutant screening. To enhance the efficiency of mutant selection, we developed a novel GCH agar medium using milk casein as the primary protein source, which allows direct visualization of protease activity based on the formation of clear halos around colonies.

Colonies grown on this medium exhibited a surrounding zone of turbidity. This turbidity, similar to that observed in the previously reported mC medium (Sasaki et al. 2025), results from the accumulation of insoluble peptide fragments generated by gingipain‐mediated casein degradation. The subsequent transition from turbidity to transparency reflects further degradation and solubilization of these fragments, indicating that the rate of transparency progression can serve as a semi‐quantitative indicator of protease activity. Using this medium, we successfully obtained four mutants with high protease activity, demonstrating the effectiveness of this screening approach. Notably, this system enables the detection of both intrinsic enzymatic enhancement and increased protease secretion.

### 4.2 Measurement of Protease Activity and Identification of Mutants

Protease activity was measured using the Red Protease Detection Kit, which has been reported to detect a broad range of protease activities (Sigma‐Aldrich Technical Note), allowing for the potential detection of previously unreported proteases. However, in this study, mutations in the *rgpA* gene were identified, suggesting that standard BANA assays commonly used for gingipain detection (Loesche et al. 1992; Sheets et al. 2005) might have been sufficient.

Next‐generation sequencing analysis revealed 27 variants relative to the reference strain (Table S1). These were used to extract mutations specific to each mutant. In the 2,6‐DAP‐derived mutants (m017 and m024), only a single mutation was detected per strain, indicating a low mutagenesis efficiency. In contrast, EMS treatment produced a sufficient number of mutations, indicating that 10 mM EMS is suitable for effective mutagenesis in *P. gingivalis*.

### 4.3 Mutations Associated With Protease Activity

The 2,6‐DAP mutants (m017 and m024) carried mutations in genes involved in iron uptake. It is known that a decrease in iron availability upregulates *rgpA* transcription in *P. gingivalis* (Tokuda et al. 1998), likely reflecting the need for gingipains to degrade transferrin and acquire free iron (Brochu et al. 2001). Therefore, in these two strains, decreased iron uptake likely led to reduced intracellular iron levels, which in turn increased *rgpA* transcription and the amount of secreted protease. In this case, the observed increase reflects the secretion level rather than intrinsic enzyme activity, demonstrating that the screening system can detect such changes.

In EMS‐induced mutants (m060, m072), multiple mutations were observed, including mutations in RgpA, one of the major proteases of *P. gingivalis*. These mutations were located within the protease activity domain (225–719 aa), suggesting that they may have directly enhanced RgpA activity.

In contrast, although certain intergenic mutations were located in putative regulatory regions, their direct contribution to the enhanced protease phenotype remains unclear based on the present analysis. Future experiments will focus on purifying RgpA from culture supernatants (Ruggiero et al. 2013) to directly assess its activity.

### 4.4 Other Mutations Affecting Protease Secretion

In mutant m060, in addition to mutations in RgpA, additional mutations were detected in PGN_RS03970 (*sprA*/*sov*), a core component of the Type IX Secretion System (T9SS), and in PGN_RS07990, a TolC‐family gene of the Type I Secretion System (T1SS) (Table 2). Since T9SS plays an important role in gingipain secretion (Sato 2021), the observed increase in protease activity in m060 is likely attributable not only to intrinsic enhancement of RgpA activity but also to improved secretion efficiency mediated by the T9SS mutation. T1SS, although not directly responsible for gingipain export, is involved in the transport of various proteins and metabolites across the outer membrane (Spitz et al. 2019). Therefore, the mutation in PGN_RS07990 may influence the overall secretion environment or cell‐surface architecture, indirectly affecting gingipain release and contributing to the increased protease activity.

In m072, a mutation was identified in PGN_RS09820, which encodes Mfa5, a component of the minor fimbriae (Table 2). Since Mfa5 is involved in cell surface architecture (Hasegawa and Nagano 2021), this mutation may have altered cell surface structures and affected T9SS function and substrate transport efficiency.

Taken together, the mutations identified in m060 and m072 suggest that the observed increase in protease activity may arise not only from changes in intrinsic enzymatic activity but also from alterations in secretion mechanisms and cell surface architecture. In particular, mutations affecting components of the Type IX Secretion System (T9SS) are likely to contribute directly to increased extracellular protease levels by enhancing secretion efficiency. In contrast, mutations associated with cell surface–related factors, such as Mfa5 and components of the Type I Secretion System (T1SS), may indirectly modulate protease activity by influencing the efficiency of secretion or release processes. Thus, the enhanced protease activity observed in these mutants is likely the result of multifactorial effects involving both enzymatic properties and changes in secretion and cell surface structure.

### 4.5 Conclusions and Future Perspectives

This study established an efficient method to select *P. gingivalis* mutants with high protease activity. We focused on linking induced mutations to changes in extracellular protease activity, and more detailed phenotypic analyses, such as biochemical characterization and fractionation of cell‐associated and secreted proteases, were not performed and remain a topic for future investigation. Given the limited number of mutants obtained, the collection and analysis of additional mutants in the future may enable the identification of amino acid regions critical for gingipain activity. Moreover, this screening system can also be applied to isolate genes involved in protease secretion efficiency.

Although the mutants identified in this study were obtained within a single strain background, comparisons with strains exhibiting different levels of virulence, such as W83, W50, and TDC60, may provide valuable context for interpreting the phenotypic consequences of identified mutations (Boyer et al. 2020; Chen et al. 2015). Such comparisons could also inform future studies, including site‐directed mutagenesis of selected loci.

These findings may contribute to the development of gingipain inhibitors (Dominy et al. 2019) as well as to the discovery of inhibitors targeting the secretion machinery. Furthermore, this method has the potential to detect the three major gingipains as well as previously unidentified proteases that are expressed at low levels under laboratory conditions, making it a promising approach for the discovery of novel proteases.

## Funding

This study was supported by internal funding from H.U. Group Research Institute G.K.

## Conflicts of Interest

The authors declare that they have no conflicts of interest to disclose.

## Supporting information




**Table S1**. List of variants in the WT strain relative to the reference genome.

**Table S2**. List of variants in the four mutant strains.

## Data Availability

The raw sequencing reads have been deposited in the NCBI Sequence Read Archive (SRA) under BioProject accession number PRJNA1423358. All relevant data are in the article and Supporting Information.
